# Conservation of Complex Nuclear Localization Signals Utilizing Classical and Non-Classical Nuclear Import Pathways in LANA Homologs of KSHV and RFHV

**DOI:** 10.1371/journal.pone.0018920

**Published:** 2011-04-29

**Authors:** Lidia Cherezova, Kellie L. Burnside, Timothy M. Rose

**Affiliations:** 1 Department of Global Health, University of Washington, Seattle, Washington, United States of America; 2 Center for Childhood Infections and Prematurity Research, Seattle Children's Research Institute, Seattle, Washington, United States of America; 3 Department of Pediatrics, University of Washington, Seattle, Washington, United States of America; University of Hong Kong, Hong Kong

## Abstract

ORF73 latency-associated nuclear antigen (LANA) of the Kaposi's sarcoma-associated herpesvirus (KSHV) is targeted to the nucleus of infected cells where it binds to chromatin and mediates viral episome persistence, interacts with cellular proteins and plays a role in latency and tumorigenesis. A structurally related LANA homolog has been identified in the retroperitoneal fibromatosis herpesvirus (RFHV), the macaque homolog of KSHV. Here, we report the evolutionary and functional conservation of a novel bi-functional nuclear localization signal (NLS) in KSHV and RFHV LANA. N-terminal peptides from both proteins were fused to EGFP or double EGFP fusions to examine their ability to induce nuclear transport of a heterologous protein. In addition, GST-pull down experiments were used to analyze the ability of LANA peptides to interact with members of the karyopherin family of nuclear transport receptors. Our studies revealed that both LANA proteins contain an N-terminal arginine/glycine (RG)-rich domain spanning a conserved chromatin-binding motif, which binds directly to importin β1 in a RanGTP-sensitive manner and serves as an NLS in the importin β1-mediated non-classical nuclear import pathway. Embedded within this domain is a conserved lysine/arginine-(KR)-rich bipartite motif that binds directly to multiple members of the importin α family of nuclear import adaptors in a RanGTP-insensitive manner and serves as an NLS in the classical importin α/β-mediated nuclear import pathway. The positioning of a classical bipartite kr-NLS embedded within a non-classical rg-NLS is a unique arrangement in these viral proteins, whose nuclear localization is critical to their functionality and to the virus life cycle. The ability to interact with multiple import receptors provides alternate pathways for nuclear localization of LANA. Since different import receptors can import cargo to distinct subnuclear compartments, a multifunctional NLS may provide LANA with an increased ability to interact with different nuclear components in its multifunctional role to maintain viral latency.

## Introduction

Kaposi's sarcoma (KS) is a multifocal vascular neoplasm that develops in conjunction with HIV infection and AIDS. Epidemiological data strongly supports the role of the human rhadinovirus, Kaposi's sarcoma-associated herpesvirus/human herpesvirus 8 (KSHV), as the causative agent of KS [1]. The majority of KS spindleoid tumor cells are latently infected with KSHV and express a limited number of KSHV proteins that are important for maintenance of the viral genome within the proliferating tumor cells [2]. The ORF73 latency-associated nuclear antigen (LANA) is a nuclear protein that is expressed in all cells that are latently infected with KSHV [3], [4], [5]. LANA functions to tether the viral episomal DNA to host-cell chromosomes by binding as a dimer to terminal repeats of the viral DNA [6], [7] and to histone 2A and 2B bound to host-cell DNA [8]. LANA also inhibits apoptosis and p53-mediated signaling [9], interacts with the retinoblastoma protein [10] and glycogen synthase kinase-3β [11], and inhibits lytic replication [12], [13]. Thus, LANA is responsible for the replication, maintenance, and persistence of the viral genome within the host cell, and promotes the survival of the infected tumor cell.

We have previously sequenced the ORF73 LANA homolog of the retroperitoneal fibromatosis-associated herpesvirus *M. nemestrina*, (RFHVMn) [14]. RFHVMn is the macaque homolog of KSHV infecting pig-tailed macaques (*M. nemestrina*), and is associated with retroperitoneal fibromatosis (RF), a KS-like tumor of macaques [15]. Like KSHV LANA, the RFHVMn LANA homolog localizes to the nucleus of transfected mammalian cells *in vitro* and to the nucleus of RFHVMn-infected RF tumor cells *in vivo* where it is believed to perform functions similar to KSHV LANA in the maintenance of viral latency [14]. A comparison of the encoded KSHV and RFHVMn LANA proteins revealed significant sequence homology, including the presence of a large internal acidic repeat region and strong sequence similarity in the N-terminal basic domain implicated in nuclear localization and chromatin binding [14].

Nuclear localization of large proteins, such as LANA, requires a nuclear localization signal (NLS) that mediates binding to members of the karyopherin family of nuclear transport proteins. The karyopherins transport NLS-containing cargo proteins through the nuclear pore into the nucleus where the cargo protein is released to function [16], [17]. Cargo release occurs by binding of RanGTP to the karyopherin transporter [18]. Since RanGTP is asymetrically distributed in the cell, with higher concentrations in the nucleus than in the cytoplasm, directed transport and release into the nucleus is achieved.

The importin β superfamily of karyopherins consists of more than 20 distinct receptors related to importin β/importin β1, the first identified receptor (see review [19]). The proteins show weak overall sequence similarity with the strongest homology in the N-terminal RanGTP binding domain. The remainder of the protein consists of 19–20 tandem HEAT repeats that have a superhelical architecture providing a versatile domain for NLS-cargo interactions. Most importin β family members, including importin β1 and β2 (transportin), bind to NLS-cargos directly, a pathway historically referred to as non-classical transport. In the more well-defined classical transport pathway, NLS-cargo proteins interact with an adapter protein belonging to the importin α family, which in turn forms a heterocomplex with importin β1 for nuclear transport [16], [20]. The importin α family consists of six related proteins in humans and five in mice that have different spatial and temporal expression patterns [21], [22]. The non-classical pathway appears to be more evolutionarily ancient and is utilized in nuclear transport of many basic cellular components, such as ribosomal proteins [23], heterogeneous nuclear ribonuclear proteins (hnRNPs) [24], [25], core histones [26], [27], [28], transcription factors [29], [30], [31], and cyclins [32].

Classical NLSs that bind to importin α consist of short stretches of basic amino acids, typically lysine-rich, which may occur as single or bipartite motifs. Monopartite motifs contain a common structure of a hexapeptide or heptapeptide with three to five basic amino acids either flanked by an N-terminal proline or glycine or containing an internal proline [17] with a general consensus sequence of K(K/R)X(K/R) [20]. Bipartite motifs have a general consensus sequence of (K/R)(K/R)X_10–12_(K/R)_3/5_ where at least 3 of 5 consecutive residues are arginine or lysine after a variable linker region [33], [34]. Structural studies have determined an optimal bipartite consensus sequence of KRX_10–12_KRRK for binding to importin α [35], [36].

Non-classical NLSs that bind directly to importin β family members are less well defined and show specificity for individual importins. Arginine is the predominant basic amino acid in these motifs, which are often much longer than the classical lysine-rich motifs. The arginine-rich NLSs of HTLV-1 Rex protein [37], HIV-1 Rev [38], [39], and HIV-1 Tat [38] bind to importin β1, independent of importin α. An extended complex lysine/arginine (Lys/Arg)-rich motif of the ribosomal protein L23a binds to importin β1, as well as importins β2, β5, and β7 [23]. A number of nuclear RNA-binding proteins have been identified that interact with importin β through arginine/glycine-rich (RG) domains [40], [41]. Proteins containing RG domains have been implicated in RNA transport due to the presence of “RGG” RNA-binding motifs and are targeted to the nucleolus [40], [42]. Non-arginine-rich NLSs have been identified that bind to importin β family members, including a lysine-rich motif in parathyroid hormone-related protein (PTHrP) [43], and a glycine-rich M9 sequence of the hnRNP A1 protein [44], [45]. Some proteins have been identified with both classical and non-classical NLS motifs that can interact directly with both importin α and importin β family members. In rare cases the same motifs bind both classes of importins, for example the adenovirus core protein pVII [46], while in other cases, like the guanine nucleotide binding protein GNL3L [47], separate motifs bind to different importins.

Previous studies have suggested that the N- and C-terminal regions of KSHV LANA each contain a putative NLS and independently localize to the nucleus [48], [49], [50]. The N-terminal NLS has been localized to an “RKRNRSP” motif located at aa 24–30 [48]. This sequence contains the K(K/R)X(K/R) consensus motif for NLS binding to importin-α. However, the KSHV NLS lacks the N-terminal or internal proline found in classical NLS motifs, as described above. Instead, it is flanked by C-terminal serine and proline residues. Similar NLSs have been identified in other nuclear proteins including the EBNA-1 protein of Epstein-Barr virus (EBV), a major latency-associated protein involved in maintenance of the EBV genome in infected cells [51]. The EBV EBNA-1 contains an NLS “EKRPRSP” matching the consensus importin-α binding motif and has been shown to bind importin α1 and importin α5 for nuclear import [52], [53]. The EBV EBNA-1 protein is considered the closest structural and functional homolog to KSHV LANA, which suggests that the conserved NLS sequences in these viral proteins may serve a common function in gammaherpesvirus latency.

Here, we report that the previously characterized “RKRNRSP” motif of KSHV LANA is not sufficient to induce nuclear localization of a fused EGFP dimer nor can it bind to members of the importin family for nuclear import. Instead, our results indicate that the “RKRNRSP” motif is part of a classical Lys/Arg-rich bipartite NLS (kr-NLS), which is conserved in the LANA homolog of macaque RFHVMn and interacts with multiple members of the importin α adapter family for nuclear import by the classical importin α/β-mediated pathway. Furthermore, this bipartite kr-NLS is embedded in a larger domain rich in arginines and glycines (rg-NLS), which interacts with importin β1 directly to utilize a non-classical nuclear import pathway. Thus, these LANA homologs have evolved a conserved mechanism for interacting with multiple importin-mediated transport pathways that allow them to traffic to the nucleus, a critical step in establishing and maintaining viral latency in an infected cell.

## Results

### The “RKRNRSP” NLS motif of KSHV LANA is not conserved in the LANA homolog of the macaque RFHVMn

**Figure 1:**
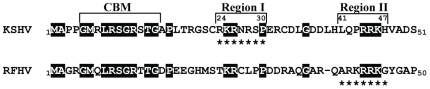
Conservation of the N-terminal domains of the ORF73 LANA homologs of KSHV and RFHVMn. The N-terminal domains of the LANA homologs of KSHV (human; NP_572129) and macaque RFHVMn (pig-tailed macaque; ABH07414) were aligned to identify conserved motifs. The previously characterized chromatin binding motif (CBM) and NLS of KSHV LANA (Region I) [8], [48], [54] and the predicted NLS motif of RFHVMn (Region II) are indicated. Critical residues and conservation in other sequences are highlighted.

Our previous studies demonstrated that ORF73 LANA of the macaque RFHVMn, like KSHV LANA, localizes to the nuclei of infected cells *in vitro* and *in vivo* [14]. To identify the NLS motif inducing nuclear localization of RFHVMn LANA, we compared the N-terminal domain of KSHV LANA containing its experimentally determined NLS motif “RKRNRSP” (aa24–30; termed Region I in this study) [48], with the corresponding domain of RFHVMn LANA. The RFHVMn LANA sequence “_24_ TKRCLPP_30_” that is positionally aligned with the “_24_ RKRNRSP_30_” of KSHV LANA contains only two of the four basic residues predicted to be functional components of the NLS (Region I, Fig. 1), suggesting that the RFHVMn sequence would not similarly function as an NLS. In comparison, the chromatin-binding motif (CBM) of KSHV LANA [8], [54], just upstream of the NLS, was strongly conserved in RFHVMn LANA with conservation of all nine critical amino acids (CBM region, Fig. 1).

### RFHVMn LANA contains a novel Lys/Arg-rich motif predicted to function as an NLS

The RFHVMn LANA sequence was analyzed using software algorithms developed to predict NLS motifs. We used both the “predictNLS” (http://cubic.bioc.columbia.edu/predictNLS) and the “PSORT” (http://psort.nibb.ac.jp/form.html) programs. “PSORT” predicted the nuclear sorting of both KSHV and RFHVMn LANA. While the “predictNLS” software was unable to identify the experimentally determined “_24_ RKRNRSP_30_” NLS motif of KSHV LANA, it identified a Lys/Arg-rich sequence “_40_ ARKRRKG_46_” in RFHVMn LANA, Region II (Fig. 1). The RFHVMn motif contained the “K(K/R)X(K/R) consensus sequence for NLS motifs interacting with the importin α adapter [20] and displayed five consecutive basic amino acids, a feature strongly predicted to cause nuclear localization. The RFHVMn motif was positioned downstream of the KSHV “RKRNRSP” motif at aa 40–46, termed Region II in this study. The KSHV LANA sequence in Region II, _41_ LQPRRKH_47_, positionally corresponding to the _40_ ARKRRKG_46_ RFHVMn motif, had a minimal conservation of three of the five basic residues “RRK” (Fig. 1). The “predictNLS” program did not identify Region II in KSHV LANA as a putative NLS even though it contained some features of a conventional NLS including four basic amino acids with an N-terminal proline residue “PRRKH” (Fig. 1).

### The _24_ RKRNRSP_30_ motif in Region I of KSHV LANA mediates nuclear localization of an EGFP fusion protein

To identify functional NLS motifs, we used an expression system that produces an N-terminal test peptide fused to the enhanced green fluorescence protein (EGFP) to determine nuclear targeting. EGFP fusions with KSHV LANA peptides were prepared containing either the Region I _24_ RKRNRSP_30_ NLS peptide (ksI_24–30_), the Region II sequence _41_ LQPRRKH_47_ (ksII_41–47_), or the entire peptide sequence from Region I to Region II (ksI+II_20–49_). An alanine and glycine substituted peptide “AGGAGAG” derived from Region II of RFHVMn LANA was used as a negative control EGFP fusion peptide. The EGFP fusion constructs were transfected into Vero cells and allowed to express EGFP for 12–18 hours, as described in Materials and Methods. The localization of EGFP fluorescence was determined by confocal microscopy.

**Figure 2:**
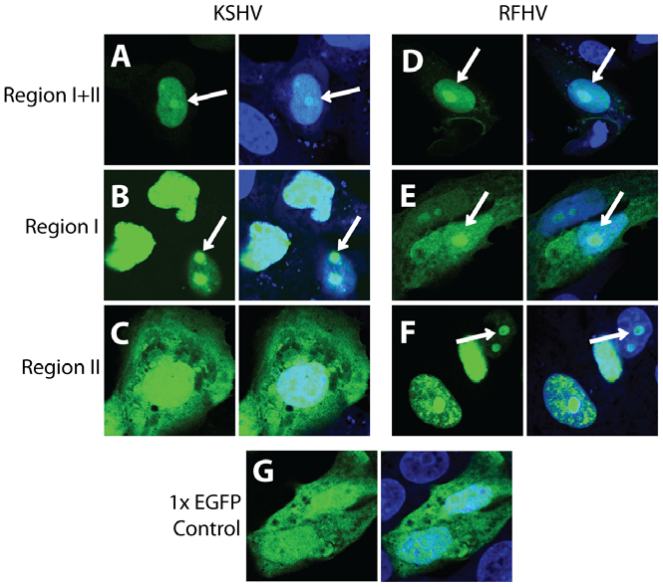
Region I _24_ RKRNRSP_30_ peptide of KSHV LANA and Region II _40_ ARKRRKG_46_ peptide of RFHVMn LANA induce the nuclear and nucleolar localization of 1×EGFP. To examine the ability of N-terminal domains of KSHV and RFHVMn LANA to induce the nuclear translocation of a heterologous protein, Vero cells were transfected with a single EGFP fused to an “AGGAGAG” control peptide (G) or the KSHV LANA N-terminal peptides ksI+II_20–49_ (Region I+II) (A), ksI_24–30_ (Region I) (B), or ksII_41–47_ (Region II) (C) or the RFHVMn LANA N-terminal peptides rfI+II_20–48_ (Region I+II) (D), rfI_24–30_ (Region I) (E) or rfII_40–46_ (Region II) (F). Cells were stained with Topro-3 to allow localization of the nuclear DNA. Left panels show EGFP fluorescence (green); right panels show the merged image of Topro-3 DNA fluorescence (blue) and EGFP (green) fluorescence. Strong cytoplasmic EGFP fluorescence was observed in C, E and G, while nuclear and nucleolar (arrows) EGFP fluorescence were observed in A, B, D, and F. The results from the cells shown in A–G are representative of the other cells in the respective fields.

The EGFP control peptide showed a diffuse localization within the cytoplasm and the nucleus (Fig. 2G), as expected for a 25 kD protein that can passively diffuse through nuclear pores [55]. In contrast, ksI+II_20–49_(EGFP), containing both Regions I and II was targeted to the nucleus, as shown by the co-localization of EGFP and Topro-3 fluorescence (Fig. 2A, EGFP (left) and Merged (right)). A sub-nuclear concentration of EGFP, characteristic of a nucleolar staining pattern, was observed in all of the transfected cells (arrow, Figure 2A). The ksI_24–30_(EGFP), containing only the _24_ RKRNRSP_30_ NLS motif of Region I, was strongly targeted to the nucleus (Fig. 2B) and gave a very distinct accumulation in specific nuclear spots, characteristic of nucleoli (arrow, Fig. 2B). The ksII_41–47_(EGFP), containing only Region II, showed a strong cytoplasmic expression of EGFP (Fig. 2C) and gave essentially the same fluorescence pattern as the control EGFP (Fig. 2G). Thus, the Region I peptide _24_ RKRNRSP_30_ is sufficient to target a single EGFP to the nucleus, confirming previous results [48].

### RFHVMn LANA contains a novel Lys/Arg-rich motif in Region II that mediates nuclear localization of an EGFP-fusion

To determine whether the RFHVMn LANA contains a functional NLS, EGFP fusion constructs were prepared with either the Region I peptide _24_ TKRCLPP_30_ (rfI_24–30_) positionally corresponding to the _24_ RKRNRSP_30_ NLS of KSHV, the putative Region II NLS peptide _40_ ARKRRKG_46_ (rfII_40–46_), or the entire peptide from Region I to Region II (rfI+II_20–48_) (see Fig. 1). Transfection of rfI+II_20–48_(EGFP) into Vero cells gave a strong nuclear EGFP fluorescence (Fig. 2D), very similar to that seen with the corresponding KSHV peptide (Fig. 2A). In contrast, the Region I peptide of RFHV (rfI_24–30_), unlike the Region I peptide of KSHV, showed minimal ability to localize EGFP to the nucleus, as significant cytoplasmic fluorescence was detected in every cell. However, some nucleolar accumulation was observed (Fig. 2E). The putative _40_ ARKRRKG_46_ NLS in Region II (rfII_40–46_), demonstrated a strong nuclear and nucleolar-like targeting of EGFP fluorescence (Fig. 2F), similar to that seen with the KSHV Region I _24_ RKRNRSP_30_ peptide (Fig. 2B). These results contrasted with the corresponding Region II peptide of KSHV, which gave a very strong cytoplasmic localization for EGFP (Fig. 2C). Thus, RFHVMn LANA contains a distinct monopartite Lys/Arg-rich motif, _40_ ARKRRKG_46_ , which functions similarly to the KSHV _24_ RKRNRSP_30_ motif. Whereas the KSHV motif is located in Region I of the LANA sequence, the RFHVMn motif is located in Region II and therefore does not appear to be evolutionarily related to the KSHV motif.

### The monopartite Lys/Arg-rich NLS motifs of KSHV and RFHVMn LANA are not sufficient for efficient interaction with members of the importin family of nuclear transporters

**Figure 3:**
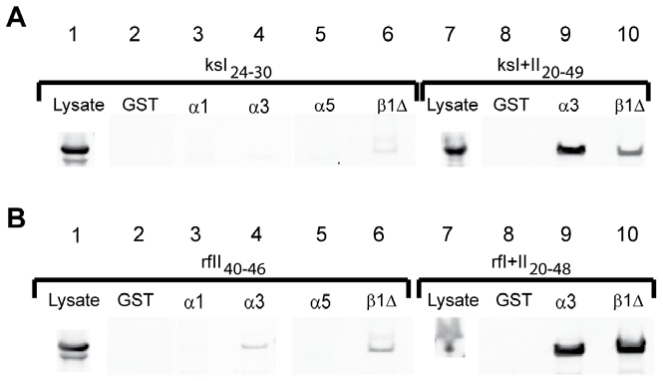
The bipartite Region I/II motifs of KSHV and RFHVMn LANA interact strongly with importin α and importin β1. To examine the ability of the KSHV and RFHVMn LANA N-terminal domains to bind to members of the importin α and β family of nuclear import receptors, Cos7 cells were transiently transfected with constructs expressing either A) ksI_24–30_ (Region I) (lanes 1–6) or ksI+II_20–49_ (Region I+II) (lanes 7–10) of KSHV LANA fused to a single EGFP or B) rfI_40–46_ (Region II) (lanes 1–6) or rfI+II_20–48_ (Region I+II) (lanes 7–10) of RFHVMn LANA fused to a single EGFP. Cell lysates were incubated with equivalent amounts of purified GST or GST-tagged members of the importin α and β family of proteins that were immobilized on glutathione-sepharose beads. The beads were washed and bound fusion proteins were eluted and analyzed by Western blot analysis using an antibody to EGFP, as described in the Materials and Methods. Pull-downs were performed with purified GST alone (Lanes 2 and 8), GST- importin α1 (Lane 3), GST- importin α3 (Lanes 4 and 9), GST- importin α5 (Lane 5), and GST-importin β1Δ_(1–462)_ (Lanes 6 and 10). As described in the text, importin β1Δ_(1–462)_ is a deletion mutant of importin β1 containing the N-terminal 462 amino acids and lacks the C-terminal domain that binds to importin α.

To delineate the mechanism of nuclear import induced by the monopartite Lys/Arg-rich NLS motifs of KSHV (Region I) and RFHVMn LANA (Region II), the ability of these motifs to interact with members of the importin family of nuclear transporters was assessed. The constructs ksI_24–30_(EGFP) and rfII_40–46_(EGFP) were transfected into Cos7 cells and the lysates were analyzed by Western blot for expressed protein using the anti-EGFP antibody. Both lysates showed abundant amounts of expressed EGFP-fusion protein (Figs. 3A,B, lane 1, respectively). Constructs expressing GST alone or different members of the importin α family as GST-tagged fusions, including importins α1, α3, and α5, were transfected into bacteria and the GST-tagged importins were purified using glutathione-sepharose beads. Similarly, a GST-tagged deletion mutant of importin β1, importin β1Δ_(1–462)_, was also purified. This deletion mutant contains aa 1–462 of importin β1, corresponding to HEAT repeats 1–11, and lacks the C-terminal domain, which interacts with importin α [32]. Equivalent amounts of glutathione-bound GST or GST-importin fusions were incubated with the Cos7 lysates, washed and analyzed by Western blot for bound EGFP-fusion proteins. No binding was detected to GST alone (Fig. 3A, lane 2). Surprisingly, no binding was detected between the _24_ RKRNRSP_30_ peptide of KSHV and either importin α1, α3, or α5 (Fig. 3A, lanes 3,4 and 5,). A very faint interaction was detected with importin β1Δ_(1–462)_ (Fig. 3A, lane 6,). No binding was detected between the Region II _40_ ARKRRKG_46_ peptide of RFHVMn and importins α1 or α5 (Fig. 3B, lanes 3 and 5). No binding was detected with GST alone (Fig. 3B, lane 2), while a small amount of binding was detected with importin α3 and β1Δ_(1–462)_ (Fig. 3B, lanes 4 and 6).

To determine whether a larger domain could induce greater importin binding, the peptides containing both Regions I and II of KSHV and RFHVMn LANA, ie. ksI+II_20–49_ and rfI+II_20–48_, respectively, were assayed for the ability to bind equivalent amounts of GST-importins α3 and β1Δ_(1–462)_. Cos7 lysates of both constructs showed abundant amounts of the EGFP fusion proteins (Figs. 3A,B, lane 7). Neither fusion protein bound to GST alone (Figs. 3A,B, lane 8). However, strong binding of ksI+II_20–49_(EGFP) and rfI+II_20–48_(EGFP) was detected to both importin α3 and importin β1Δ (Fig. 3A,B, lanes 9 and 10, respectively).

### The Region I/II bipartite NLS of KSHV LANA is required to mediate nuclear translocation of a double EGFP fusion protein

Although the Region I monopartite _24_ RKRNRSP_30_ peptide of KSHV was able to localize EGFP to the nucleus (Fig. 2B), no significant interactions were detected with different members of the importin family, raising questions regarding the mechanism of nuclear localization of these constructs. Proteins that are smaller than 40 kDa are able to enter the nucleus by passive diffusion through the nuclear pore complex, whereas proteins that are larger rely on transport proteins [56]. Since EGFP is 25 kDa and diffuses into the nucleus [55], we compared the ability of the monopartite Region I and bipartite Region I+II of KSHV LANA to promote nuclear translocation of a 56 kDa double EGFP dimer fusion protein (2×EGFP). The 2×EGFP fusion protein is above the size-limit expected for passive diffusion through the nuclear pore and has been used as an alternative to single EGFP for nuclear localization [57], [58], [59]. In addition, we examined whether the EGFP constructs would co-localize with either of the nucleolar proteins, nucleolin or B23.1, fused to the red fluorescent protein (RFP) [60], to investigate the putative nucleolar targeting of EGFP constructs in Figure 2.

**Figure 4:**
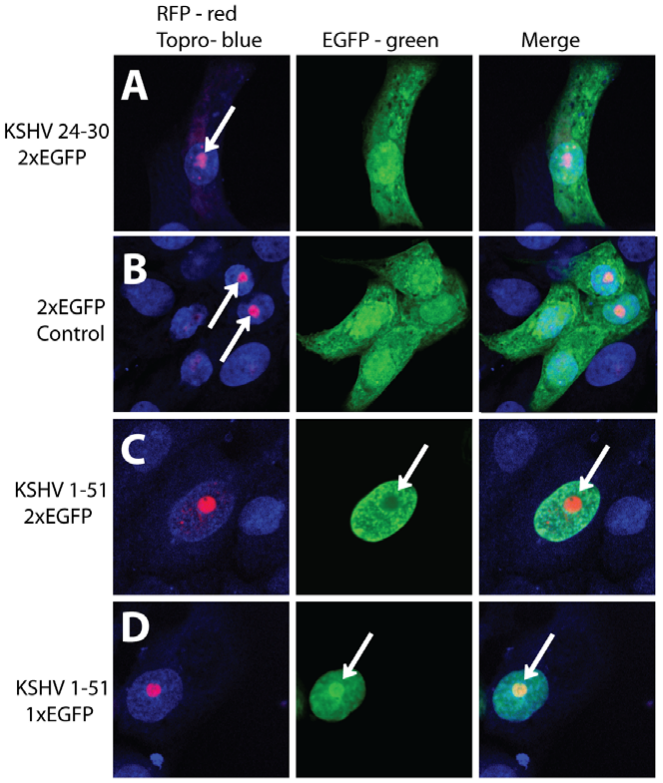
The bipartite Region I/II of KSHV LANA is required for nuclear localization of 2×EGFP, a double EGFP fusion. In order to compare the ability of KSHV LANA domains to induce the translocation of single EGFP or double EGFP fusions and examine potential nucleolar accumulation, Vero cells were co-transfected with a construct expressing the B23.1 nucleolar protein fused to RFP and constructs expressing either A) the Region I motif (KSI_24–30_) fused to a double EGFP (2×EGFP), B) 2×EGFP alone, C) the N-terminal 51 amino acids of KSHV LANA fused to 2×EGFP or D) the N-terminal 51 amino acids of KSHV LANA fused to 1×EGFP. Cells were stained with Topro-3 to allow localization of the nuclear DNA. Left panels show the merged B23.1(RFP) (red) and DNA Topro-3 (blue) fluorescence, detecting nucleolar and nuclear fluorescence, respectively. The middle panels show the EGFP fluorescence (green) and arrows indicate the nucleolar accumulation of EGFP (D) and the nucleolar exclusion of EGFP (C). The right panels show the merged B23.1(RFP), Topro-3 and EGFP fluorescence. Co-localization of nucleolar RFP and EGFP was detected in D. The lack of nucleolar co-localization of RFP and EGFP was detected in C.

Vero cells were double transfected with plasmids expressing the B23.1-RFP fusion protein and the monopartite Region I peptide KSHV 24–30 fused to 2×EGFP. A plasmid expressing 2×EGFP alone was used as a control. The B23.1-RFP red fluorescence accumulated in 1–2 discreet spots in cell nuclei, consistent with a nucleolar location (Fig. 4A,B, left panels, arrows). Fluorescence from the 2×EGFP control was spread throughout the cell showing a strong cytoplasmic and nuclear accumulation (Fig. 4B, middle and right panels), similar to that seen with the 1×EGFP control (Fig. 2G). In contrast to the results obtained with the single EGFP (Fig. 2B), the Region I KSHV 24–30 fused to 2×EGFP showed strong cytoplasmic staining (Fig. 4A, middle and right panels), equivalent to that seen with the 2×EGFP control plasmid (Fig. 4B, middle and right panels). Thus, the monopartite Region I KSHV 24–30 peptide showed no ability to induce nuclear or nucleolar localization of the larger 2×EGFP fusion. In contrast, the first 51 amino acids of KSHV LANA containing the Region I and II bipartite NLS induced the complete nuclear localization of 2×EGFP (Fig. 4C middle and right panels). This construct was excluded from the nucleolar region where B23.1-RFP was localized (arrows, Fig. 4C, middle and right panels). Interestingly, full-length KSHV LANA is also excluded from the nucleolus in similar conditions [14]. The first 51 amino acids of KSHV LANA were also able to strongly localize a single EGFP (1×EGFP) fusion protein to the nucleus (Fig. 4D, middle and right panels), similar to that seen with the truncated KSHV peptide 20–49 containing only the Region I and II bipartite NLS (Fig. 2A). In both of these cases, the single EGFP concentrated in an obvious nuclear spot, which co-localized with the nucleolar protein B23.1 (arrows, Fig. 4D right panel). Similar results were obtained with RFP-nucleolin (data not shown).

### The Region I/II bipartite NLS motifs of KSHV and RFHVMn LANA bind to multiple members of the importin α family

**Figure 5:**
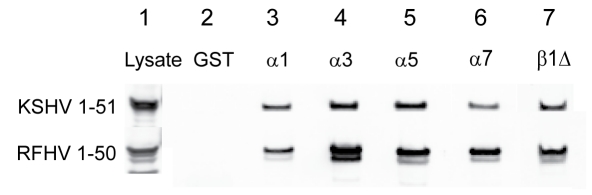
The N-terminal domains of KSHV and RFHVMn LANA containing the chromatin binding motif and the bipartite Region I/II NLS interact strongly with multiple importins. The ability of the N-terminal domains of KSHV and RFHVMn LANA to bind to importin β1 and different members of the importin α family of adaptor proteins was examined. Cos7 cells were transiently transfected with constructs expressing the N-terminal domains of either KSHV (KS_1–51_) or RFHV (RF_1–50_) fused to 2×EGFP. Cell lysates (Lane 1) were incubated with equivalent amounts of purified GST (lane 2) or GST-tagged importins α1 (lane 3), α3 (lane 4), α5 (lane 5), α7 (lane 6), or β1Δ_(1–462)_ (lane 7) immobilized on glutathione-sepharose beads and bound EGFP was detected by Western analysis.

To examine the ability of the Region I/II bipartite NLS motif of KSHV and RFHV to bind to members of the importin family, plasmids expressing EGFP fused to the N-terminal 51 and 50 amino acids of KSHV and RFHV, respectively, (KSHV_1–51_(EGFP) and RFHV_1–50_(EGFP)) were transfected into Cos7 cells and cell lysates were prepared. Western blot analysis revealed substantial amounts of the EGFP-fusion proteins (Fig. 5, lane 1). Cell lysates were incubated with equivalent amounts of glutathione-bound GST or GST-importin fusion proteins, as described above. Neither EGFP fusion bound to GST alone (Fig. 5, lane 2). Strong binding of KSHV_1–51_(EGFP) and RFHV_1–50_(EGFP) was detected to importins α1, α3, α5, α7 (Fig. 5, lanes 3,4,5 and 6) with α3 showing the most robust binding. Both constructs also bound strongly to importin β1Δ_(1–462)_ (Fig. 5, lane 7). Since the importin β1Δ_(1–462)_ deletion mutant is unable to interact with importin α, the binding of the KSHV and RFHVMn LANA constructs to importin β1 was direct.

### KSHV LANA binding to importin β1 is inhibited by RanGTP

**Figure 6:**
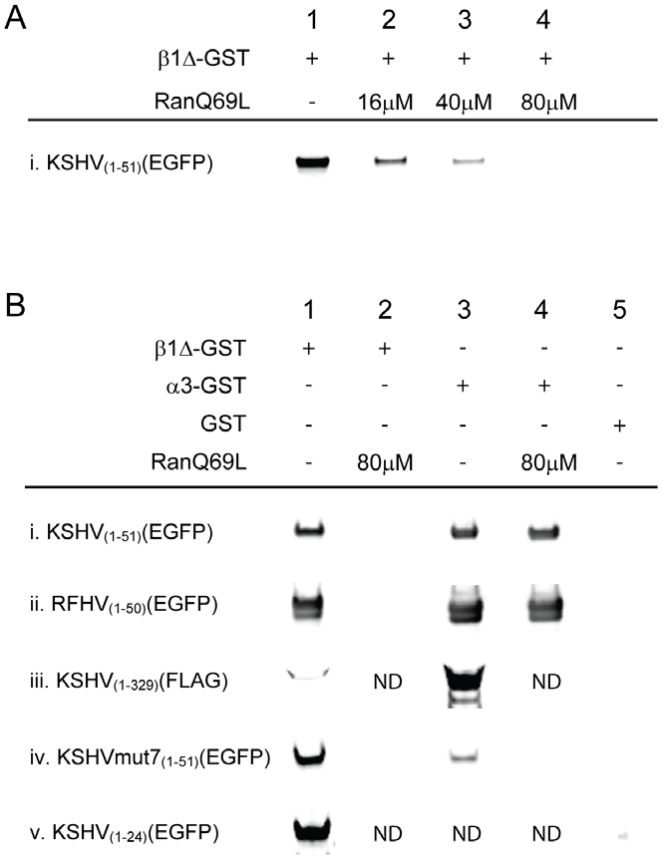
RanGTP dependency of KSHV and RFHVMn LANA-importin interactions. A) The specificity of the interaction between the KSHV LANA N-terminal domain and importin β1 was examined using a constitutively active RanGTP mutant, RanQ69L, which specifically dissociates NLS cargo bound directly to importin β1 (see text). Cos7 cells were transiently transfected with a construct expressing the N-terminal domain of KSHV (KS_1–51_) fused to 2×EGFP. GST-tagged importin β1Δ_(1–462)_ immobilized on glutathione-sepharose beads was incubated with the Cos7 cell lysates and then treated with different amounts of purified RanQ69L that had been loaded with GTP. Bound EGFP was detected by Western analysis. B) The specificity of binding of different KSHV and RFHVMn LANA constructs to importin α and β was examined using RanQ69L. Cos7 cells were transiently transfected with constructs expressing i) the N-terminal 51 amino acids of KSHV LANA (KS_1–51_) fused to 2×EGFP, ii) the N-terminal 50 amino acids of RFHVMn LANA (RF_1–50_) fused to 2×EGFP, iii) the FLAG-tagged N-terminal 329 amino acid domain of KSHV LANA (KSHV_1–329_), iv) the N-terminal 51 amino acids of KSHV LANA containing alanine substitutions of the Region I and Region II Lys/Arg motifs (KSHV mut7) fused to 2×EGFP, v) the N-terminal 24 amino acids of KSHV LANA (KS_1–24_) fused to 1×EGFP. Cell lysates were incubated with either equivalent amounts of purified GST-tagged importin β1Δ_(1–462)_ (lanes 1 and 2), α3 (lanes 3 and 4) or GST alone (lane 5) immobilized on glutathione-sepharose beads. The beads were then incubated with either 80 µM RanQ69L loaded with GTP (lanes 2 and 4) or no RanQ69L (lanes 1,3 and 5). Bound proteins were detected by Western analysis using anti-EGFP (rows i,ii, iv and v) or anti-FLAG (row iii). ND = not determined.

To determine the specificity of the interactions between the KSHV LANA N-terminal domain and importin β1Δ_(1–462)_, we investigated whether the interaction was sensitive to competition by the small GTPase Ran. Ran loaded with GTP dissociates NLS cargoes from importin β-related import receptors [61]. We used the constitutively-active RanQ69L mutant that is deficient in the ability to hydrolyze GTP [62]. 6×His RanQ69L was purified and loaded with GTP, as described in Materials and Methods. GST- importin β1Δ_(1–462)_ immobilized on glutathione beads was incubated with lysates of Cos7 cells expressing KSHV_1–51_(EGFP), washed and further incubated with increasing amounts of RanQ69L-GTP. Bound EGFP was detected by Western analysis. Strong binding of the KSHV_1–51_(EGFP) to importin β1Δ_(1–462)_ was detected in the absence of RanQ69L-GTP (Fig. 6Ai, lane 1), as shown previously (Fig. 5, lane 7). This interaction was inhibited by RanQ69L-GTP in a dose-dependent manner, with all binding competed at 80 µM (Fig. 6Ai, lane 4), demonstrating that the binding of KSHV_1–51_(EGFP) to importin β1 was not only direct but specific.

### KSHV and RFHV LANA binding to importin α is direct and not sensitive to RanGTP

The ability of the KSHV and RFHVMn LANA N-terminal constructs to bind directly to importin β1 raised the possibility that the interactions with importin α detected in the GST-pulldown experiments could have been mediated by endogenous importin β1 in the Cos7 lysates, in an indirect manner. While Ran-GTP dissociates interactions between importin β and NLS-containing cargos, it does not affect binding of cargos to importin α. We therefore examined the ability of KSHV_1–51_(EGFP) and RFHV_1–50_(EGFP) to bind to importin α, using importin α3, which showed the most robust binding in Figure 5. Both constructs were expressed in Cos7 cells, incubated with equivalent amounts of GST-tagged importin α3 or β1Δ_(1–462)_ immobilized on glutathione beads, and then incubated with 80 µM RanQ69L-GTP. KSHV_1–51_(EGFP) interacted strongly with importin β1Δ_(1–462)_ (Fig. 6Bi, lane 1) and importin α3 (Fig. 6Bi, lane 3). While the interaction with importin β1Δ_(1–462)_ was completely inhibited by RanQ69L-GTP (Fig. 6Bi, lane 2), the interaction with importin α3 was not inhibited (Fig. 6Bi, lane 4). Similar results were obtained with the RFHV_1–50_(EGFP) construct (Fig. 6Bii).

We also investigated the ability of the full-length N-terminal domain of KSHV LANA to bind to importin family members. A FLAG-tagged C-terminal truncation of KSHV LANA, containing the 329 aa N-terminal domain (KSHV_1–329_; FLAG-LANA_N_ [49]) was expressed in Cos7 cells and incubated with equivalent amounts of GST-tagged importin α3 or β1Δ_(1–462)_ immobilized on glutathione beads. Bound protein was detected by Western blot analysis using an anti-FLAG antibody. The full-length N-terminal domain of KSHV LANA interacted strongly with importin α3 (Fig. 6Biii, lane 3) and weakly with importin β1Δ (Fig. 6Biii, lane 1).

### Alanine substitutions of the basic Lys/Arg residues in the Region I/II bipartite NLS of KSHV eliminates binding to importin α, but does not eliminate binding to importin β1 or nuclear targeting of 2×EGFP fusions

**Figure 7:**
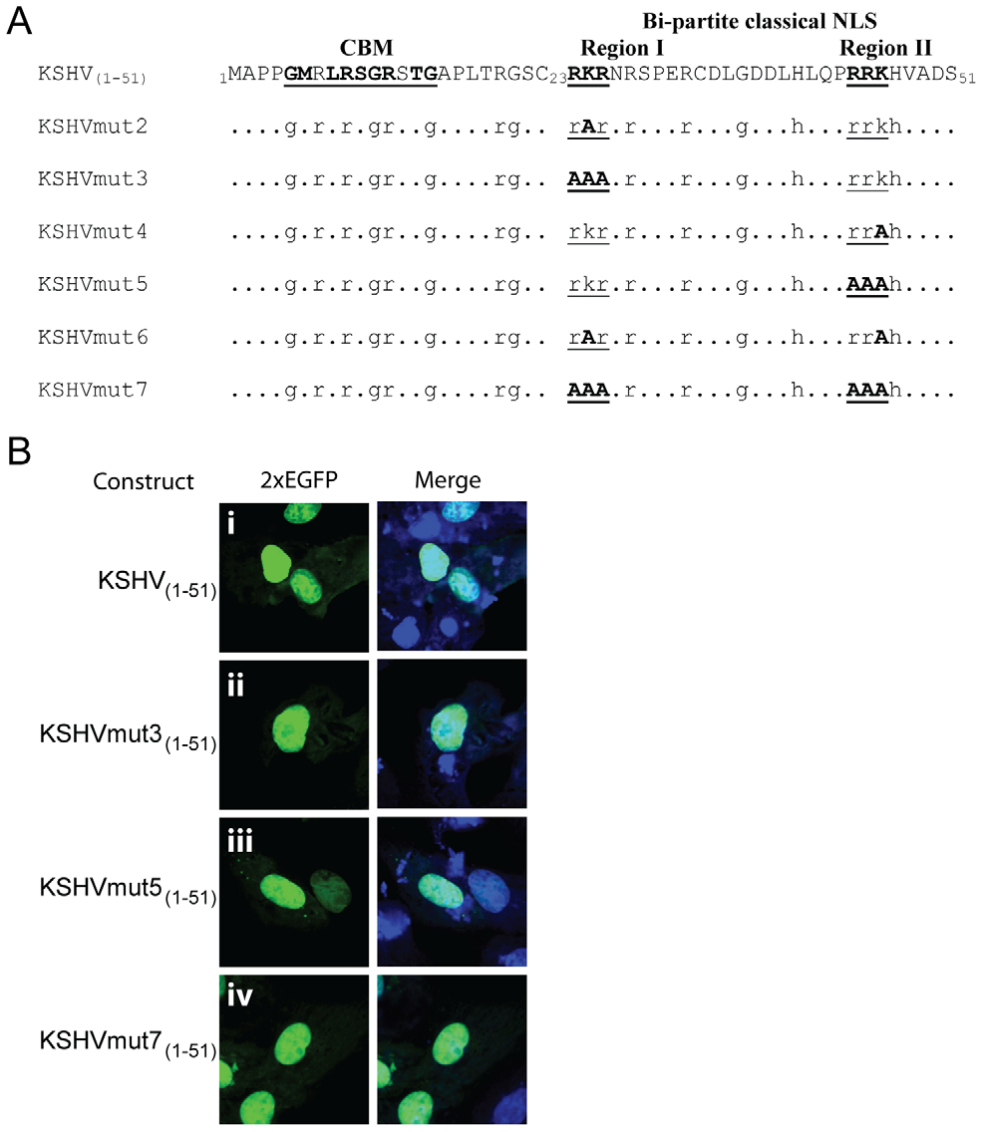
Alanine substitution of the Lys/Arg motifs in the bipartite classical NLS of KSHV LANA abrogates importin α binding, but does not alter nuclear localization of 2×EGFP or binding to importin β1. A) To examine the importance of the Lys/Arg motifs in the bipartite NLS of KSHV LANA, constructs were prepared with various alanine substitutions for the Lys/Arg cores within the N-terminal 51 amino acids of KSHV LANA. The chromatin binding motif (CBM) and bipartite NLS regions are indicated. The underlying glycine, arginine and histidine residues within the N-terminal domain are shown. B) Vero cells were transfected with 2×EGFP fusion constructs containing the different alanine substitutions in the context of the N-terminal 51 amino acids and fluorescence was monitored by confocal microscopy. Left panel, EGFP fluorescence; right panel Topro-3 DNA and EGFP merged fluorescence. Strong nuclear fluorescence was observed for the wild type construct and all of the alanine substitution mutants. The 2×EGFP control showed strong cytoplasmic fluorescence (data not shown), identical to that in Fig. 4B. The ability of the KSHV mut7, with all six Lys/Arg residues substituted by alanine, to bind to importin α3 and β1Δ_(1–462)_ was examined in Figure 6B, row iv.

In order to examine the dependence of the putative classical bipartite NLS of KSHV LANA on the clusters of basic residues in Region I and II, 2×EGFP fusion constructs were prepared containing various alanine substitutions for the lysines and arginines in the background of the 51 amino acid N-terminal peptide. Alanine substitutions for the single lysine residues in Regions I and II or triple substitutions of the lysines and two flanking arginine residues were made in both regions (Fig. 7A). The KSHV_1–51_ wild-type (2×EGFP) or alanine-substituted peptide fusion constructs were transfected into Cos7 cells and examined by confocal microscopy. Surprisingly, none of the single or triple alanine substitutions in either Region I, Region II or both Region I and II eliminated the ability of the 51 amino acid peptide to target 2×EGFP to the nucleus (Fig. 7B (mutants 3, 5 and 7) and data not shown (mutants 2, 4 and 6)).

To determine the mechanism for nuclear targeting of the KSHV N-terminal domain lacking the Lys/Arg clusters in Region I and II, the KSHVmut7, containing alanine substitutions for both lysine residues and four arginine residues, was transfected into Cos7 cells and lysates were incubated with equivalent amounts of GST or GST-importins immobilized on glutathione beads. The beads were washed and bound EGFP was detected by Western analysis. No binding was detected with GST alone (Fig. 6Biv, lane 5). Weak binding was detected with importin α3 (Fig. 6Biv, lane 3), however, this binding was competed by RanQ69L-GTP (Fig. 6Biv, lane 4) indicating that the binding was indirect, mediated through endogenous importin β1 present in the Cos7 cell extract. These results show that the alanine substitutions in KSHV mut7 eliminated the ability of KSHV 1–51 peptide to bind directly to importin α. This demonstrates that the Lys/Arg residues in Region I and II constitute a classical bipartite Lys/Arg-rich NLS (kr-NLS) that is necessary for importin α interaction.

In contrast, strong binding of KSHVmut7 was detected with importin β1Δ_(1–462)_ (Figure 6Biv, lane 1). This binding was competed by RanQ69L-GTP (Fig. 6Biv, lane 2) showing that the interaction with importin β1 was direct and specific, and occurred in the absence of the bipartite kr-NLS motif. Examination of the sequence of the KSHVmut7 construct revealed the presence of a substantial number of arginine residues interspersed with glycine residues throughout the 51 aa N-terminal domain (see Fig. 7A). Arginine/glycine (Arg/Gly)-rich sequences have been identified as NLS motifs that utilize “non-classical” pathways for nuclear transport, in which direct binding of the cargo NLS to members of the importin β family of proteins is independent of the importin α adapter [37], [38]. Thus, the classical bipartite kr-NLS in KSHV LANA, which was disrupted by the alanine substitutions in KSHVmut7, is embedded within a larger Arg/Gly-rich motif (rg-NLS) that can interact with importin β1 directly and induce nuclear localization through the non-classical import pathway.

A previous study had noted that the first 24 amino acids of the KSHV LANA N-terminus could direct nuclear localization of a single EGFP, although this was thought to be due to the diffusion of EGFP into the nucleus and sequestration by the chromatin-binding motif [54]. We obtained the KSHV_1–24_(EGFP) construct used in this study and expressed it in Cos7 cells. The cell lysate was incubated with GST-tagged importin β1Δ_(1–462)_ immobilized on glutathione beads. Western blot analysis of the bound proteins revealed substantial amounts of bound KSHV_1–24_(EGFP) (Fig. 6Bv, lane 1). This was similar to the binding detected between importin β1Δ_(1–462)_ and the ksI+II_20–49_ peptide (Fig. 3, lane 10). Much weaker, but detectable interactions were also seen between importin β1Δ_(1–462)_ and the ksI_24–30_ peptide, itself (Fig. 3, lane 6). These results suggest that the non-classical rg-NLS that binds importin β1 extends across the whole Arg/Gly-rich 51 amino acid N-terminal domain of KSHV LANA, including the chromatin-binding motif. The results obtained with the RFHVMn LANA N-terminal domain peptides, rfI+II_20–48_ (Fig. 3, lane 10) and rfI_40–46_ (Fig. 3, lane 6) support the conclusion that RFHVMn LANA also contains an extended N-terminal non-classical rg-NLS that binds directly to importin β1.

## Discussion

We previously cloned and characterized ORF73 LANA of RFHVMn, the macaque homolog of KSHV, in order to compare the sequence with KSHV LANA and identify evolutionarily conserved functional features [14]. A comparison of the N-terminal region revealed an exact conservation of critical residues in the KSHV chromatin-binding motif, aa 5–17 [8], [54]. However, minimal sequence similarity was found with the adjacent KSHV NLS motif “RKRNRSP”, aa24–30, determined previously [48]. This previous study reported that 1) deleting aa1–32 of KSHV LANA abrogated nuclear localization, 2) aa1–32 could restore nuclear localization to a KSHV LANA deletion mutant, 3) aa1–32 could target EGFP to the nucleus, and 4) the _24_ RKRNRSP_30_ peptide could induce nuclear translocation of β-galactosidase [48].

To verify the functionality of the _24_ RKRNRSP_30_ peptide, we prepared a series of EGFP constructs containing different regions of the N-terminal domain of KSHV LANA. Our studies showed that the _24_ RKRNRSP_30_ peptide, termed Region I in this study, could induce the nuclear translocation of EGFP, while a downstream peptide _41_ LQPRRKH_47_, termed Region II in this study, could not. Because of concern in the literature that a single EGFP with a molecular weight of 25 Kd could diffuse through the nuclear pores [58], [59], we also constructed a series of fusions with a double EGFP construct, having a molecular weight greater than 50 kDa. In contrast to the single EGFP results, we found that the _24_ RKRNRSP_30_ peptide could not induce nuclear localization of the 2×EGFP fusion. Since Piolot et al. found that _24_ RKRNRSP_30_ was able to induce nuclear localization of β-galactosidase, another large protein, we examined the insertion site of the RKRNRSP peptide in the β-galactosidase expression vector used in their study to try to resolve this discrepancy. Our analysis revealed that _24_ RKRNRSP_30_ had been adventitiously inserted into β-galactosidase downstream and upstream of two additional lysine residues yielding the sequence KvpRKRNRSPvpK. Since basic residues play an important role in nuclear localization, the ability of _24_ RKRNRSP_30_ to induce nuclear localization of β-galactosidase may have been due to additional basic residues derived from the vector insertion site.

We further examined the ability of the _24_ RKRNRSP_30_ peptide to induce nuclear localization by investigating its ability to bind to different members of the importin α and β family of nuclear transport receptors. We were unable to detect binding of _24_ RKRNRSP_30_ to any of the importin α family members tested and only very weak binding to importin β1. Thus, our studies indicated that _24_ RKRNRSP_30_, by itself, was not sufficient for efficient interaction with the nuclear import machinery nor was it able to induce the nuclear localization of a cargo protein too large to easily diffuse across the nuclear pore. Although we detected strong nuclear localization of _24_ RKRNRSP_30_ (1×EGFP) and accumulation in nucleoli, confirmed by co-localization of B23.1-RFP and nucleolin-RFP, we believe this resulted from diffusion into the nucleus and retention through the peptide interactions with other cellular nuclear and/or nucleolar constituents. We did not see any significant differences in the accumulation of 1×EGFP and 2×EGFP fusion protein controls throughout the cell nucleus and cytoplasm after 24 hours. However, we did detect differences in the ability of the 1×EGFP and 2×EGFP to localize to the nucleus and nucleolus when fused to different targeting peptides. Our data suggests that care should be taken when interpreting nuclear localization studies with a single EGFP.

A previous study noted the presence of Lys/Arg-rich motifs in Regions I and II of KSHV LANA, suggestive of a bipartite NLS [54]. Our studies showed that constructs containing both Regions I and II induced the nuclear localization of 2×EGFP and strongly interacted with importin α1, α3, α5, and α7 family members, in a RanGTP-insensitive manner. Neither Region I or II alone was sufficient to induce nuclear localization of 2×EGFP or bind to importin α, substantiating the bipartite nature of the NLS. Our results are compatible with the study of Piolot et al. [48], which showed that deletion of the first 32 amino acids of KSHV LANA abrogated nuclear localization, since this would disrupt the bipartite NLS. Our studies also demonstrated that the RFHVMn LANA NLS is bipartite and utilizes Lys/Arg-rich motifs in Regions I and II for efficient binding to importin α1, α3, α5, and α7 family members. Weak binding to α3 was also detected with the RFHVMn Region II peptide “_40_ ARKRRKG_46_” alone, which was predicted to function as a monopartite NLS in our bioinformatic analysis. This peptide has an additional lysine residue compared to the Region I _24_ RKRNRSP_30_ peptide of KSHV, which may have slightly increased its affinity to importins.

**Figure 8:**
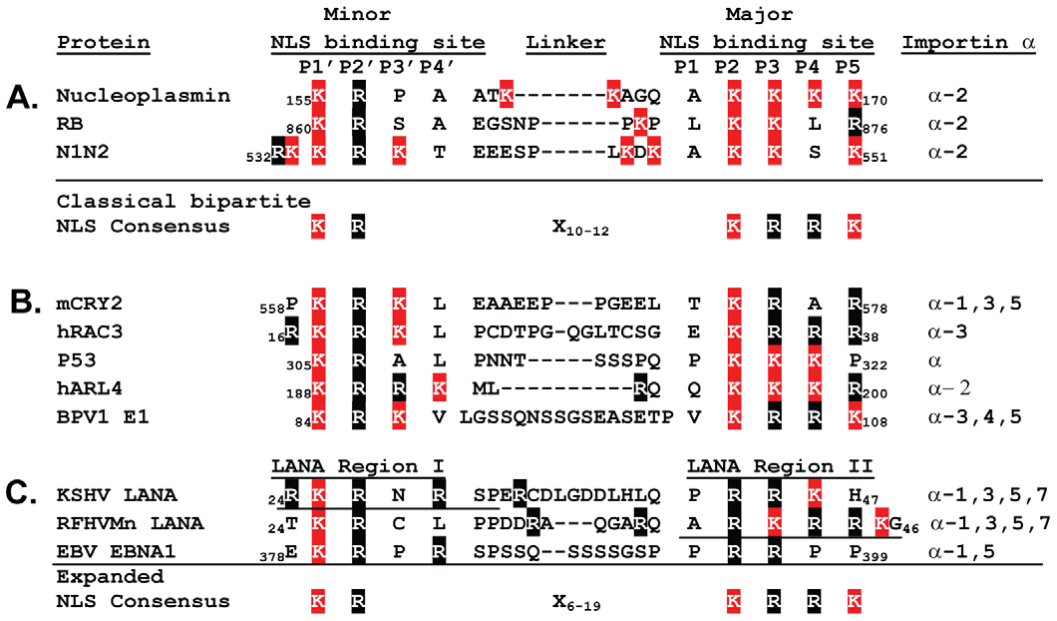
Classical bipartite NLS motifs that bind to importin α. A) The binding of classical bipartite NLS motifs to specific binding pockets of importin α, determined structurally. The classical bipartite NLS consensus proposed previously [36] is indicated. B) Consensus alignment of the bipartite NLS motifs of additional proteins that functionally interact with importin α family members. C) Consensus alignment of the classical bipartite NLS motifs of KSHV and RFHVMn LANA. The alignment of a putative bipartite NLS motif in EBV EBNA-1 is indicated. An expanded consensus sequence proposed for classical bipartite NLS motifs that interact with importin α family members is shown. References include: Nucleoplasmin [35]; Retinoblastoma protein (RB) [73]; Chromatin assembly factor N1N2 [36]; mammalian circadian clock component (mCRY2) [74]; nuclear receptor coactivator hRAC3 [75]; p53 [76]; ADP ribosylation factor hARL4 [77]; bovine papillomavirus type 1 E1 (BPV1 E1) [78]; EBV EBNA1 [52], [53].

Alignment of the KSHV and RFHVMn N-terminal LANA sequences revealed a conserved “KR” motif in Region I and a conserved “RRK” motif in Region II separated by a 16–17 amino acid linker sequence. This closely matches the (KRX_10–12_KRRK) consensus sequence for classical bipartite NLSs that interact with importin α. Studies have shown that the N-terminal “KR” motif of bipartite NLSs binds the P1′ and P2′ positions of the minor binding site in importin α, while the larger “KRRK” motif binds the P2–P5 positions of the major binding site in importin α [36] (Fig. 8A). The NLS motifs of other proteins reacting with importin α have also been determined and alignment of the sequences revealed further conservation of the bipartite consensus model (Fig. 8B). The KSHV and RFHVMn LANA bipartite kr-NLS motifs fit this model very closely, although the KSHV NLS has a larger number of basic residues in Region I that would interact with the minor binding site, while RFHVMn NLS has a larger number of basic residues in Region II that would interact with the major binding site (Fig. 8C). Interestingly, EBV EBNA-1, a distantly-related homolog of LANA has been shown to bind to importins α1 and α5 [52], [53]. The EBNA-1 NLS has been localized to the “_378_ EKRPRSP_384_” motif, which is very similar to the _24_ RKRNRSP_30_ of KSHV LANA. Analysis of the flanking sequences in EBNA-1 revealed a downstream “PRR” motif that is also found in the Region II basic cluster of KSHV LANA, indicating that the EBNA-1 NLS also matches the bipartite consensus (Fig. 8C). The requirement for the bipartite NLS in EBNA-1 for binding to importin α remains to be determined. The linker region of the KSHV LANA bipartite kr-NLS is 16 residues long, while those of RFHVMn LANA and EBV EBNA-1 are both 15 residues long. Although the original bipartite consensus sequence predicted a linker length of 10–12 residues [36], our studies and those of others have identified bipartite NLS motifs interacting with importin α with linker regions ranging from 6–19 residues in length, suggesting the need for an expanded consensus of “KRX_6–19_KRRK” (Fig. 8C).

To examine the role of the KSHV LANA “KR” and “RRK” motifs in binding to importin α and inducing nuclear localization, we prepared alanine substitutions of these residues within the KS_1–51_ peptide fused to 2×EGFP. Unexpectedly, all of these constructs, including KSHV mut7, which contained alanine substitutions of the “RKR” motif in Region I and the “RRK” motif in Region II, were still able to efficiently induce nuclear localization of 2×EGFP. In GST-pull down experiments, the alanine substitutions in KSHV mut7 abrogated its ability to bind to importin α demonstrating a requirement for the “RKR” and “RRK” motifs for a functional classical bipartite NLS that binds importin α. Previous studies have shown that deletion of the N-terminal 30 aa, which removes the Region I “RKR” motif disrupting the Region I+II classical bipartite NLS, prevents nuclear localization of full-length LANA [48].

Our studies showed that the KSHV mut7 was able to strongly bind importin β1Δ_(1–462)_, as did the N-terminal 24 aa (KS_1–24_), the 30 aa across Region I and II (KSI+II_20–49_) and the 51 N-terminal aa (KS_1–51_). In all cases, the binding was inhibited by RanQ69L-GTP, demonstrating its specificity. The intact 329 aa N-terminal domain of KSHV LANA was also able to bind importin β1Δ_(1–462)_, albeit more weakly. The β1Δ_(1–462)_ mutant is a truncation that contains only the N-terminal domain and HEAT repeats 1–11 of the full-length importin β1 [32]. The truncation mutant maintains the Ran-GTP binding domain [63] but is unable to bind to importin α, which interacts with HEAT repeats 7–19 in the C-terminal domain [64]. Therefore, our results suggest that the nuclear localization of KSHV mut7 occurred through direct binding to importin β1, independent of importin α, through the non-classical nuclear transport pathway. Many proteins with arginine-rich non-classical NLSs bind to the C-terminal HEAT repeats of importin β1 and compete with importin α binding, including Tat [38], Rex [37], and Nab2 [65]. In contrast, the ribosomal protein L23a [23] and the parathyroid hormone-related protein PTHrP both bind to HEAT repeats 1–11 of importin β1, similar to KSHV and RFHVMn LANA. PTHrP has been shown to bind to importin β1 simultaneously with importin α [66], suggesting that LANA could function similarly.

**Figure 9:**
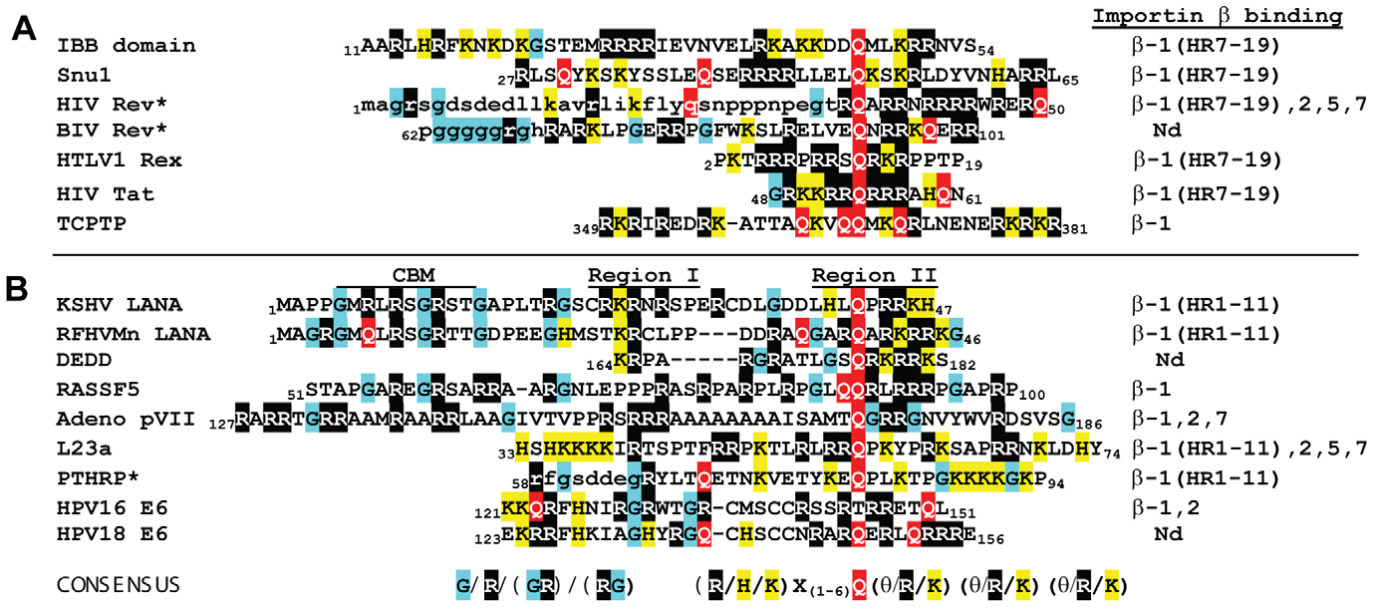
Non-classical NLS motifs that bind to importin β1. Alignment of non-classical NLS motifs that interact with importin β1 in the A) C-terminal IBB binding domain, HEAT repeats 7–19: importin β binding site (IBB) of importin α [64]; Snurportin 1 (Snu1) [79]; HIV1 Rev [38], [39]; BIV Rev (importin β binding uncharacterized, aligned by homology to HIV1 Rev [60]; HTLV1 Rex [37]; HIV Tat [38]; T-cell protein tyrosine phosphatase (TCPTP)(binding domain uncharacterized) [80] or B) N-terminal domain, HEAT repeats 1–11: KSHV and RFHVMn LANA (this study); Death effector domain-containing protein (DEDD) (importin β binding uncharacterized, but accumulates in the nucleolus, like other importin β-binding proteins) [81]; Ras-associated tumor suppressor (RASSF5) (binding domain uncharacterized) [67]; Adenovirus core protein pVII (specific β1 binding site not determined) [46]; ribosomal protein L23a [23]; parathyroid related protein (PTHRP) [43], [66]; Human papillomavirus 16 (HPV16) E6 protein (the N-terminal “KKQR” is required for binding) [69]; Human papillomavirus 18 (HPV18) E6 protein (importin β binding not determined, aligned by homology to HPV16) [69]. A consensus sequence motif “(R/H/K)-X_(1–6)_-Q-(θ/R/K)-( θ/R/K)- (θ/R/K)”, where θ is a hydrophobic residue, was identified in arginine-rich non-classical NLSs that bind importin β1 directly. Proteins that bind preferentially to the N-terminal domain of importin β1 through HEAT repeats 1–11 contained additional glycine and arginine residues with “GR” and “RG” dipeptide motifs. *flanking sites upstream of identified NLS motifs are shown in lowercase.

Sequence analysis of the KS_1–51_ N-terminal domain revealed the presence of an extended stretch of arginines and glycines, even after the alanine substitutions of the Lys/Arg motifs in Regions I and II in KSHV mut7. Similar Arg-rich and Arg/Gly-rich sequences are characteristic of non-classical NLS motifs in proteins that bind importin β1 directly, including the tumor suppressor RASSF5 [67], the adenovirus core protein PVII [46], the parathyroid related protein (PTHrP) [43], the transcription factors Smad-3 [68] and SREBP-2 [30], the hnRNP-like protein Nab2 [65], the retroviral proteins Rev and Tat in HIV-1 [38] and Rex in HTLV [37], the ribosomal proteins L23a and L5 [23] and cyclin B1 [32]. Although no obvious consensus sequence has yet been determined for non-classical NLSs that bind to importin β1, we identified a conserved glutamine residue that is flanked by an extended region of basic residues both upstream and downstream. A consensus motif of (R/H/K)X_(1–6)_ **Q**(θ/R/K)(θ/R/K)(θ/R/K), where θ represents a hydrophobic amino acid, is present in KSHV and RFHVMn LANA and other importin β1-binding proteins (Fig. 9). In proteins, like KSHV and RFHVMn LANA, that bind to the N-terminal domain of importin β1 (HEAT repeats 1–11) (Fig. 9B), an extended region of glycines and arginines containing characteristic “RG” and “GR” dipeptide motifs were identified upstream of the conserved glutamine residue. A close amino acid similarity was detected between the importin β1-binding domains of the tumor suppressor RASSF5 and KSHV and RFHVMn LANA (Fig. 9B). However, RASSF5 lacks the Lys/Arg-rich classical bipartite NLS that allows KSHV and RFHVMn LANA to also interact directly with importin α. The conserved glutamine and flanking basic residues were also detected in proteins that bind the C-terminal HEAT repeats 7–19, including the importin β-binding (IBB) domain of importin α, although the presence of upstream Arg/Gly residues was not as prevalent (Fig. 9A). The N-terminal domain of importin β1 containing HEAT repeats 1–11 is considered to be representative of an ancestral nuclear import receptor [66]. This suggests that the modern importin β1 structure evolved through gene duplication providing additional NLS binding sites in the C-terminal HEAT repeats that also maintained a conserved NLS specificity.

**Figure 10:**
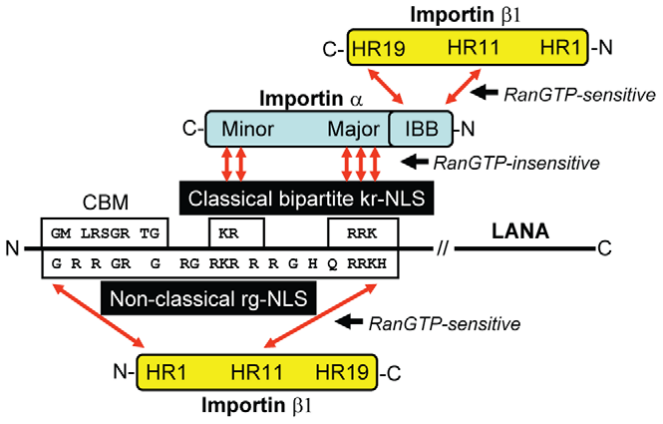
Summary of importin binding by the N-terminal KSHV LANA NLS. Residues conserved between KSHV and RFHVMn LANA within the N-terminal chromatin binding motif (CBM) and the classical bipartite Lys/Arg-rich kr-NLS are indicated above the line. Residues considered to be important within the extended non-classical Arg/Gly-rich rgNLS, including the conserved glutamine are shown below the line. The RanGTP-insensitive binding of the bipartite KSHV kr-NLS to the minor and major NLS binding sites within importin α is depicted. The RanGTP-sensitive binding of the IBB domain of importin α to the C-terminal HEAT repeats of importin β1, a critical step in the importin α/β-mediated classical nuclear import pathway (see text), is shown. The direct, RanGTP-sensitive binding of the N-terminal HEAT repeats of importin β1 to the non-classical rg-NLS of KSHV LANA, a critical step in the importin α-independent non-classical nuclear import pathway mediated by importin β1 is also depicted (see text).

We show that the LANA homologs of KSHV and the macaque RFHVMn have a conserved classical bipartite kr-NLS that binds to multiple importin α isoforms in a RanGTP-insensitive manner and induces nuclear localization of a large heterologous protein, 2×EGFP (see Fig. 10). The 329 aa N-terminal domain of KSHV LANA binds strongly to importin α suggesting that nuclear import of the intact LANA occurs primarily via the classical pathway. The classical bipartite NLS is embedded within a larger extended Arg/Gly-rich sequence (rg-NLS) that can interact with importin β1 independently in a RanGTP-sensitive manner and induce nuclear localization via the non-classical pathway (see Fig. 10). Previous studies have shown that deletion of either the N-terminal 30 aa of LANA, which eliminates the Region I cluster of basic amino acids and disrupts the classical bipartite kr-NLS, or deletion of the N-terminal 50 aa of LANA, which completely eliminates the kr-NLS, prevents nuclear localization of full-length LANA [48]. These deletions also disrupt or eliminate the extended rg-NLS that mediates interaction with importin β1, and thus would prevent nuclear import of the full-length LANA through either classical or non-classical pathways. Deletion of the N-terminal 22 amino acids of KSHV LANA, which removes the chromatin-binding motif and a large portion of the rg-NLS that we have shown to mediate interaction with importin β1 (Fig. 6Bv) but not affect the bipartite kr-NLS, does not block nuclear localization of the full-length LANA [50], [54]. Finally, Piolot et al., also showed that deletion of only Region I, aa24–30, eliminated nuclear localization of full-length LANA. These studies provide further evidence that full-length LANA primarily utilizes the classical nuclear import pathway, although the impact of these deletions on the rg-NLS and utilization of the non-classical pathway is not known.

Other examples of multivalent NLS domains include the adenoviral protein pVII, which contains three NLS regions that bind to importin α, importin β1, importin β2 and importin β7 [46], and HPV16 E6 oncoprotein, which interacts independently with importin α, importin β1 and importin β2, in both classical and non-classical nuclear import pathways [69]. Other proteins, such as histones H2A and H2B [28] and the viral protein Rev [70] interact with importin β and other importin β family members, but do not interact with importin α. The ability of LANA to interact with multiple importin family members could reflect an enhanced ability to access or subvert the cellular nuclear transport machinery. The multiple binding sites could increase the affinity of LANA to the importin α/β complex providing preferential usage of the transport machinery. Alternatively, LANA could utilize multiple import pathways for differential localization within sub-nuclear compartments.

It is of interest to note that many of the proteins that interact directly with importin β1 in the non-classical transport pathway localize to the nucleolus and shuttle between the nucleus and cytoplasm (see references in the Legend to Figure 9). Our studies using single EGFP fusions demonstrated a strong nucleolar accumulation induced by the monopartite LANA NLS domains of KSHV (_24_ RKRNRSP_30_) and RFHVMn (_40_ ARKRRKG_46_) suggesting an underlying ability to interact with nucleolar components. However, the full-length LANA is actively excluded from the nucleolus (see for example [14]). We observed that the intact 329 aa N-terminal domain of LANA interacted preferentially with importin α and showed only weak interaction with importin β1. This suggests that the rg-NLS in full-length LANA is masked, possibly through interaction of the embedded bipartite kr-NLS with importin α. Thus, under normal conditions, LANA would be translocated to the nucleus via the classical importin α/β mediated pathway, which results in exclusion of LANA from the nucleolus.

Since KSHV LANA plays a major role in both maintenance of viral latency by tethering the viral episome to host chromatin and tumor induction by interacting with nuclear proteins to subvert normal host cell function, its transport to the nucleus is paramount for its function. Our studies delineating the mechanism of nuclear transport of LANA suggests that it has evolved to contain compensatory nuclear localization signals to ensure nuclear transport in infected cells by multiple pathways. Our results provide the basis for developing approaches to block nuclear import of LANA, which could prevent KSHV-induced malignancies and alter the old adage that “Herpesvirus infections are forever”.

## Materials and Methods

### Sequence analysis

The sequences of the ORF73 LANA homologs from KSHV and RFHVMn were aligned using ClustalW. In order to identify possible nuclear localization sequences, the LANA sequences were analyzed using the web-based “predictNLS” (http://cubic.bioc.columbia.edu/predictNLS) and “PSORT” (http://psort.nibb.ac.jp/form.html) programs.

### Construction of LANA NLS-EGFP fusion vectors

To test the ability of different LANA peptides to function in nuclear localization, oligonucleotides encoding different putative nuclear localization signals were inserted upstream of the sequence encoding EGFP in the pEGFP-N2 vector (Clontech). The plasmids were constructed by annealing pairs of oligonucleotides (labeled a and b) for each test construct (see Table 1). The annealed oligonucleotides were ligated into HindIII and KpnI digested pEGFP-N2. The following EGFP fusion constructs were confirmed through sequencing: Control plasmid: AGGAGAG; KSHV plasmids: ksI_24–30_, ksII_41–47_, ksI+II_20–49_; RFHVMn plasmids: rfI_24–30_, rfII_40–46_, rfI+II_20–48_ (see Table 1). The EGFP KSHV_1–51_ and RFHV_1–50_ LANA NLS constructs were created by annealing the following oligos from Table 1: (KSHV) ks1–51 a1–4, and b1–3; (RFHV) rf 1–50 a1,2, and b1,2. The annealed oligos were ligated into HindIII/KpnI digested pEGFP-N2. To create alanine substitution mutants of KSHV LANA 1–51, oligos bearing point mutations at positions 25 (a3a,b2a), 24–26 (a3b,b2b), 46 (a4a,b1a), 44–46 (a4b,b1b) were annealed with ks1–51 a1,a2 and b3 and ligated into HindIII/KpnI digested pEGFP-N2 vector.

**Table 1:** Oligonucleotide Summary.

Oligonucleotide	Sequence ( 5′-3′)
**AGGAGAG** **a**	AGCTGCCACCATGGGAATTCAAGCTGGAGGCGCTGGCGCAGGAGGGTAC
**AGGAGAG** **b**	CCTCCTGCGCCAGCGCCTCCAGCTTGAATTCCCATGGTGGC
**ksI_24–30_a**	AGCTGCCACCATGGGAATTCAAGCTAGAAAAAGGCGAAAAGGAGGGTAC
**ksI_24–30_b**	CCTCCTTTTCGCCTTTTTCTAGCTTGAATTCCCATGGTGGC
**ksII_41–47_a**	AGCTGCCACCATGGGAATTCAACTACAACCGCGAAGGAAGCATGGGTAC
**ksII_41–47_b**	CCATGCTTCCTTCGCGGTTGTAGTTGAATTCCCATGGTGGC
**ksI+II_20–49_a1**	AGCTGCCACCATGAGAGGAAGTTGTAGGAAACGAAACAGGTCTCCGGAAAGATGTGAC
**ksI+II_20–49_a2**	CTTGGCAATGACCTACATCTACAACCGCGAAGGAAGCATGTCGCGTAC
**ksI+II_20–49_b1**	GCGACATGCTTCCTTCGCGGTTGTAGATGTAGGTCATTGCCAAGGTCACATCTTTCCG
**ksI+II_20–49_b2**	GAGACCTGTTTCGTTTCCTACAACTTCCTCTCATGGTGGC
**rfI_24–30_a**	AGCTGCCACCATGGGAATTCAAACAAAAAGATGCCTGCCGCCGGGGTAC
**rfI_24–30_b**	CCCGGCGGCAGGCATCTTTTTGTTTGAATTCCCATGGTGGC
**rfII_40–46_a**	AGCTGCCACCATGGGAATTCAAGCTAGAAAAAGGCGAAAAGGAGGGTAC
**rfII_40–46_b**	CCTCCTTTTCGCCTTTTTCTAGCTTGAATTCCCATGGTGGC
**rfI_20–32_a**	AGCTGCCACCATGGGACATATGTCTACAAAAAGATGCCTGCCGCCGGATGACGGGTAC
**rfI_20–32_b**	CCGTCATCCGGCGGCAGGCATCTTTTTGTAGACATATGTCCCATGGTGGC
**rfI+II_20–48_a1**	AGCTGCCACCATGGGACATATGTCTACAAAAAGATGCCTGCCGC
**rfI+II_20–48_a2**	CGGATGACCGCGCCCAGGGCGCGCGTCAAGCTCGCAAAAGGCGAAAAGGGTACGGGTAC
**rfI+II_20–48_b1**	CCGTACCCTTTTCGCCTTTTGCGAGCTTGACGCGCGCCCTGG
**rfI+II_20–48_b2**	GCGCGGTCATCCGGCGGCAGGCATCTTTTTGTAGACATATGTCCCATGGTGGC
**ks 1–51 a1**	AGCTTGCCACCATGGCGCCCCCGGGAATGCGCCTGAGGTC
**ks 1–51 a2**	GGGACGGAGCACCGGCGCGCCCTTAACGAGAGGAAGTTGT
**ks 1–51 a3**	AGGAAACGAAACAGGTCTCCGGAAAGATGTGACCTTGGCGA
**ks 1–51 a3a**	AGGGCACGAAACAGGTCTCCGGAAAGATGTGACCTTGGCGA
**ks 1–51 a3b**	GCTGCAGCAAACAGGTCTCCGGAAAGATGTGACCTTGGCGA
**ks 1–51 a4**	TGACCTACATCTACAACCGCGAAGGAAGCATGTCGCCGACTCCGGGTAC
**ks 1–51 a4a**	TGACCTACATCTACAACCGCGAAGGGCACATGTCGCCGACTCCGGGTAC
**ks 1–51 a4b**	TGACCTACATCTACAACCGGCAGCGGCACATGTCGCCGACTCCGGGTAC
**ks 1–51 b1**	CCGGAGTCGGCGACATGCTTCCTTCGCGGTTGTAGATGTAGGTCATCGCCAAGGTCACAT
**ks 1–51 b1a**	CCGGAGTCGGCGACATGTGCCCTTCGCGGTTGTAGATGTAGGTCATCGCCAAGGTCACAT
**ks 1–51 b1b**	CCGGAGTCGGCGACATGTGCCGCTGCCGGTTGTAGATGTAGGTCATCGCCAAGGTCACAT
**ks 1–51 b2**	CTTTCCGGAGACCTGTTTCGTTTCCTACAACTTCCTCTCGTTAAGGGCG
**ks 1–51 b2a**	CTTTCCGGAGACCTGTTTCGTGCCCTACAACTTCCTCTCGTTAAGGGCG
**ks 1–51 b2b**	CTTTCCGGAGACCTGTTTGCTGCAGCACAACTTCCTCTCGTTAAGGGCG
**ks 1–51 b3**	CGCCGGTGCTCCGTCCCGACCTCAGGCGCATTCCCGGGGGCGCCATGGTGGCA
**rf 1–50 a1**	AGCTGCCACCATGGCCGGCCGTGGAATGCAACTACGGTCCGGCCGTACCACCGGCGACCCC
**rf 1–50 a2**	GGATGACCGCGCCCAGGGCGCGCGTCAAGCTCGCAAAAGGCGAAAAGGGTACGGAGCACCAGGGTAC
**rf 1–50 b1**	TCTTCGGGGTCGCCGGTGGTACGGCCGGACCGTAGTTGCATTCCACGGCCGGCCATGGTGGC
**rf 1–50 b2**	CCTGGTGCTCCGTACCCTTTTCGCCTTTTGCGAGCTTGACGCGCGCCCTGGGCGCGGTCATCCGGCG

The p2×EGFP vector, expressing a double EGFP fusion, was created by PCR amplification of the EGFP gene with the following primers: pEGFPapaIa (5′- ATCGGGCCCGCCACCATGGTGAGCAAG-3′) and pEGFPBamHIb (5′-TCACGGATCCCCTTGTACAGCTCGTCCATGC-3′) that contained ApaI and BamHI restriction sites. The resulting DNA product was digested with ApaI and BamHI and inserted in-frame and upstream of the existing EGFP gene within the pEGFP-N2 vector. To create 2×EGFP fused to KSHV LANA 24–30 or 1–51, the oligos ksI 24–30 a and b or KS 1–51 a1–4, b1–3 (Table 1) were inserted into HindIII/KpnI digested p2×EGFP.

### Cells

Vero and Cos7 cells were obtained from the ATCC and used in nuclear localization and importin binding studies, respectively. Cos7 cells were cultured in D-MEM complete media at 37°C, while Vero cells were cultured in GlutaMAX high glucose D-MEM complete (Gibco) (D-MEM complete: 10% Cosmic-Calf Serum (Hyclone), 100 U/ml penicillin, 100 µg/ml streptomycin, 1.0 mM HEPES, 0.1 mM non-essential amino acids, 1.0 mM sodium pyruvate).

### Confocal immunofluorescence analysis

Vero cells (1–2.5×10^4^) were plated onto 17 mm diameter circular spots drawn with the ImmEdge pen (Vector Labs) on 60 mm dishes. In some cases, the cell populations were synchronized by serum starvation for 22–24 hours. The different pEGFP expression constructs (0.5 µg) were transfected using either Lipofectamine 2000 (Invitrogen) or TransIt LT1 (Mirus) per manufacturer's instructions. Cells were incubated at 37°C for 12–24 hrs and treated with 100 µg/ml of cycloheximide (Sigma) for 2–4 hrs prior to processing for microscopy to allow post-translational nuclear accumulation of the EGFP fusion proteins. Cells were fixed with 8% para-formaldehyde, permeabilized and analyzed using confocal fluorescence microscopy (Zeiss), as described previously [14]. Nuclei were visualized using Topro-3 DNA staining (1∶100)(Molecular Probes). Nucleolar localization was confirmed using nucleolar proteins nucleolin and B23.1 fused to the red fluorescent protein (RFP), RFP-nucleolin and RFP-B23.1 [60] (kindly provided by D. Archambault).

### Antibodies

Mouse monoclonals anti-EGFP JL-8 (Clontech) and FlagM2 (Sigma) and the DyLight 680 anti-mouse secondary antibody (Rockland) were utilized at a 1∶10000 dilution in Western blot analysis.

### GST–pull down assays

Plasmids expressing the GST-tagged importins α1, α3, α5, and α7 were a gift from R. Fagerlund and were described previously [71], [72]. The plasmid expressing the GST-tagged importin β1 N-terminal fragment (1–462) was a gift from S. Kornbluth, and was described previously [32]. GST-tagged importin constructs were expressed in E.coli HB101 cells under IPTG induction for 5 hours. Bacterial pellets were resuspended in buffer L [72] and the lysates were sonicated and cleared by centrifugation at 15,000 rpm for 5 min. Bacterial expression of the GST-importin fusion proteins was confirmed by SDS-PAGE/Western blot analysis using an antibody to GST (data not shown). Cleared lysates containing the expressed GST-importin fusion proteins were incubated with glutathione-sepharose 4 fast flow beads (GE Healthcare) at 4°C overnight. The GST-importin-bound sepharose beads were washed three times and an aliquot was analyzed by SDS-PAGE/Western blot to quantitate the levels of bacterial-expressed GST-fusion proteins bound to the sepharose beads (Figure S1). Cos7 cells were transiently transfected with pEGFP NLS constructs using TransIt LT1 transfection reagent (Mirus) per manufacturer's instructions. Cells were harvested 24 hours post transfection in 1% NP40 lysis buffer (50 m M Tris, 150 mM NaCl, 5% glycerol, 1% NP-40) with complete inhibitor tablet cocktail (Roche). Equivalent amounts of GST or GST-tagged importins immobilized on glutathione-sepharose beads were incubated with Cos7 cell lysates for 2 hours at 4°C. The beads were washed with 1% NP40 cell lysis buffer and bound proteins were solubilized in 2× NuPage LDS reducing sample buffer, boiled and loaded on 4–12% Bis-Tris Nupage gels (Invitrogen).

### RanQ69L-GTP competition assay

pET28a-6×His-RanQ69L was a gift from D. Forbes (San Diego, CA). 6×His tagged RanQ69L was purified on Talon resin, eluted with imidazole elution buffer (Qiagen), desalted using a Centricon 10 concentrator column and loaded with GTP as previously described [62]. The 6×His-RanQ69L-GTP was desalted again and the protein concentration was determined using the microBCA assay. GST-tagged importins immobilized on glutathione-sepharose beads were incubated with cell lysates of Cos7 overexpressing LANA NLS constructs for 2 hours at 4°C. The beads were washed with cell lysis buffer and suspended in 1×PBS. Different amounts of 80 µM 6×His-RanQ69L-GTP were added to half of the beads and PBS was added to the other half. The beads were incubated for 30 minutes at room temperature and washed with 1×PBS. Bound material was eluted with SDS-Page loading buffer and analyzed by immunoblotting using the anti-EGFP antibody.

## Supporting Information

Figure S1 **Preparation of GST-importin-sepharose beads.** GST and GST-importin fusion constructs were expressed in E. coli HB101 under IPTG induction, as described in Materials and Methods. Bacterial lysates were sonicated and cleared by centrifugation. Cleared lysates containing expressed GST or GST-importin fusion proteins were incubated with glutathione-sepharose beads. The beads were washed and an aliquot was analyzed by SDS-PAGE/Western blot to quantitate levels of bacterial-expressed GST-fusion proteins bound to the sepharose beads using an antibody to GST. Lane 1 – GST alone; Lane 2 - GST-α1 importin; Lane 3 - GST-α3 importin; Lane 4 - GST-α5 importin; Lane 5 GST-α7 importin; Lane 6 - GST- β1Δ_(1–462)_ importin. Bead volumes containing equivalent amounts of GST or GST-importin fusions, estimated from the Western blot, were used in the GST-pull down experiments.
(TIF)
