# Supplementary figures and images for: Conservation of Complex Nuclear Localization Signals Utilizing Classical and Non-Classical Nuclear Import Pathways in LANA Homologs of KSHV and RFHV
Source: PLoS One. 2011 Apr 29;6(4):e18920. doi: 10.1371/journal.pone.0018920 (PMC3084728; doi:10.1371/journal.pone.0018920)

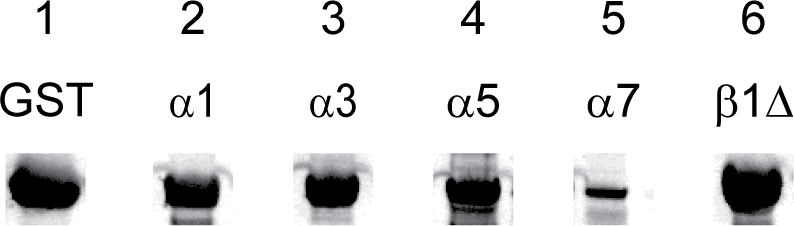

Supplement: Figure S1 — Preparation of GST-importin-sepharose beads. GST and GST-importin fusion constructs were expressed in E. coli HB101 under IPTG induction, as described in Materials and Methods. Bacterial lysates were sonicated and cleared by centrifugation. Cleared lysates containing expressed GST or GST-importin fusion proteins were incubated with glutathione-sepharose beads. The beads were washed and an aliquot was analyzed by SDS-PAGE/Western blot to quantitate levels of bacterial-expressed GST-fusion proteins bound to the sepharose beads using an antibody to GST. Lane 1 – GST alone; Lane 2 - GST-α1 importin; Lane 3 - GST-α3 importin; Lane 4 - GST-α5 importin; Lane 5 GST-α7 importin; Lane 6 - GST- β1Δ(1–462) importin. Bead volumes containing equivalent amounts of GST or GST-importin fusions, estimated from the Western blot, were used in the GST-pull down experiments. (TIF) [file pone.0018920.s001.tif]
